# Prevention in Hepatology

**DOI:** 10.3390/jpm14020132

**Published:** 2024-01-23

**Authors:** Ana-Maria Muñoz-Restrepo, Maria-Cristina Navas, Jimmy Daza, Marcos Girala, Ezequiel Ridruejo, Guido Gerken, Andreas Teufel

**Affiliations:** 1 Department of Medicine II, Division of Hepatology, Medical Faculty Mannheim, Heidelberg University, 68167 Mannheim, Germany; ana.munoz10@udea.edu.co (A.-M.M.-R.); jimmy.daza@medma.uni-heidelberg.de (J.D.); 2 Grupo Gastrohepatología, Facultad de Medicina, Universidad de Antioquia, UdeA, Calle 70 # 52-21, Medellin 050010, Colombia; maria.navas@udea.edu.co; 3 Department of Hepatology, Universidad Nacional de Asunción, San Lorenzo 111421, Paraguay; marcosgirala@yahoo.com; 4 Hepatology Section, Department of Medicine, Center for Medical Education and Clinical Research, Norberto Quirno CEMIC, Buenos Aires 1431, Argentina; eridruejo@cemic.edu.ar; 5 Department of Gastroenterology, University Hospital Essen, 45147 Essen, Germany; gugerken@icloud.com; 6 Clinical Cooperation Unit Healthy Metabolism, Center for Preventive Medicine, and Digital Health (CPD), Medical Faculty Mannheim, Heidelberg University, 69117 Mannheim, Germany

**Keywords:** liver disease, hepatology, prevention, management

## Abstract

The prevention of liver disease has improved significantly in the last few decades, to the point that it can now be considered a true success story. The wide variety of interventions, including comprehensive vaccination strategies, novel medications, lifestyle changes, and even preventive surgeries, have reduced the morbidity and mortality of chronic liver diseases. However, the prevalence of chronic liver diseases is increasing worldwide. Currently, fatty liver disease alone is estimated to be present in as much as 30% of the adult population. Furthermore, there is a trend towards increasing incidences of chronic hepatitis B, and a global lack of success in efforts to eliminate chronic hepatitis C. Thus, improving and efficiently rolling out existing and successful prevention strategies for chronic liver diseases will play an essential role in healthcare throughout the upcoming decades. In this review, we summarize the current options and concepts for preventing chronic liver diseases, highlight their limitations, and provide an outlook on probable future developments to improve awareness, integrated care, and the analysis of big data.

## 1. Background

Globally, chronic liver disease (CLD) imposes a substantial health burden, resulting in approximately two million deaths yearly (accounting for 3.5% of global mortality). Half of these deaths are attributed to Hepatocellular Carcinoma (HCC), and the other half to complications of cirrhosis [1]. A major issue is that cirrhosis often goes undetected in many patients until complications and liver cancer develop, making it the eleventh leading cause of death. Furthermore, the global health burden of CLD is expected to continue to increase, with a consistent 5% rise observed since 2000 [2].

Cirrhosis also ranks among the leading causes of disability-associated life years (DALYs) among individuals aged 50 to 74 years old [3]. The economic burden of chronic liver diseases (CLDs) is often underestimated, though some studies have tried to estimate these costs. For instance, in the case of HCC, the annual cost per patient in the United States increased in 2010, ranging from USD 133,000 for stage zero to USD 467,000 for stage D based on the Barcelona Clinic Liver Cancer Criteria (BCLC) [4].

As for the underlying causes, the estimated total cost for chronic hepatitis C (CHC) before the introduction of direct-acting antivirals (DAAs) was USD 10.6 billion, while non-alcoholic steatohepatitis (NASH) incurred a cost of USD 103 billion annually. Furthermore, the estimated 3-year healthcare cost per patient who underwent liver transplantation (LT) was USD 539,955 [5] and costs were constantly rising over the years [6].

Hence, the prevention of liver disease and its complications holds paramount importance both from a medical and economic perspective, benefiting not only patients, but also society at large.

## 2. Diverse Etiologies, Common End Stage

All liver diseases share a common end-stage outcome: fibrosis and cirrhosis, which can lead to various complications, including liver failure, hepatocellular carcinoma (HCC), and esophageal variceal bleeding, among others. Inflammation is the primary mechanism driving this progression, followed by parenchymal necrosis, activated fibrogenesis, angiogenesis, and profound vascular changes [7].

An imbalance between pro-fibrogenic and anti-fibrogenic mechanisms in the liver can result in excessive extracellular matrix production and alterations in the hepatic angioarchitecture [8]. The risk of developing these complications is influenced by several factors, including lifestyle modifications such as alcohol cessation, weight loss, and the management of infections (e.g., through hepatitis B vaccination and inflammation control) [2].

While the prevalence and incidence of common liver diseases vary significantly across the globe, the most widespread causes of cirrhosis worldwide include chronic alcohol consumption, metabolic-associated fatty liver disease (the revised term for non-alcoholic fatty liver disease), chronic viral hepatitis (with a particular emphasis on B and C), and autoimmune factors. It is important to note that a single patient may have more than one contributing cause, potentially accelerating the progression of the disease, even beyond what the presence of individual diseases and comorbidities would suggest [2].

Over the past two decades, efforts to assess chronic liver diseases (CLDs) have often concentrated on their consequences, particularly fibrosis, rather than their root causes. Thus, the need to raise public awareness of the different causes of fibrosis and cirrhosis—including fatty liver disease and alcohol consumption—and a shift of the management strategies towards preventive medicine as opposed to merely treating the complications are long overdue, especially considering that plenty of evidence suggests that early detection and preventive care could alter the future of the over two million patients who succumb annually to chronic liver diseases [9,10,11].

## 3. Hepatitis B Virus (HBV)

Among the causes of cirrhosis, hepatitis B is a life-threatening liver infection and a major global health problem caused by the hepatitis B virus (HBV). It can lead to chronic infection, primarily associated with vertical transmission in African countries and sexual transmission in Western countries [12,13]. In 2015, 3.5% of the world’s population was living with this chronic infection, with 68% in the Western Pacific and African regions. In Europe, chronic hepatitis B (CHB) affects 15 million people and leads to 56,000 deaths annually [14].

The primary goal of HBV infection treatment is to achieve a functional cure, measured by the long-term loss of hepatitis B surface antigen (HBsAg) with or without serocoversion and undetectable HBV DNA after therapy interruption. However, despite the availability of various treatments and prolonged patient care, less than 5% of individuals were HBsAg negative after 12 months of treatment [14]. The risk of up to 40% cirrhosis development in untreated patients underscores the need to promote and extend prevention programs, particularly through vaccination [2].

Several risk factors contribute to the progression of HBV infection into HCC. Viral factors such as persistent positive serum HBsAg are highly significant, as shown by a prospective study conducted in Taiwan involving 22,707 individuals, which demonstrated a relative risk of 0.66 among HBsAg+ [15,16,17]. Another study reported that, in HBsAg−-positive individuals, the joint presence of HBeAg—a marker for high HBV replication levels—increased the risk of HCC three- to sixfold compared to the HBeAg-negative population [18]. Additionally, in individuals with CHB, serum HBV DNA levels seem to predict higher rates of HCC, as patients with high counts (>10^4^ copies/mL) face an increased risk of HCC in the long-term follow-up [19]. Moreover, factors such as lifestyle are also important to consider, as chronic alcohol consumption, exposure to aflatoxin and smoking have also shown an increase in HCC risk [15,16,17].

The risk of developing chronic HBV infection in susceptible individuals depends mainly on the age of acquisition, with a decreasing tendency as the age of infection increases [20]. Among the crucial risk factors to evaluate in primary prevention, the maternal effect stands out. Neonates born to HBsAg+ mothers have a 30-fold higher risk of developing HCC compared to those born to HBsAg− mothers [21]. Hence, practical strategies for hepatitis B infection control and prevention are crucial from an early age. Infant vaccination stands out as the most effective strategy to achieve the goal of the ‘Elimination of viral hepatitis by 2030,’ which is one of the international Sustainable Development Goals.

Since 1991, the WHO has recommended the inclusion of HBV vaccination in national immunization programs [22]. By the end of 2019, the HBV vaccine had been introduced nationwide in 189 (97%) countries. However, vaccination coverage varies significantly across WHO regions. The regions of the Western Pacific, the Americas, and Southeast Asia exceed the global average, while the European, Eastern Mediterranean, and African regions fall below it [23].

In Western countries endemic for hepatitis B, the typical schedule for the HBV vaccine includes a monovalent birth dose administered within 24 h of birth to all newborns, effectively preventing perinatal transmission. This is followed by three doses of an HBV vaccine at 2, 4, and 6 months [2]. While immunization schedules may vary globally, all have demonstrated the ability to induce seroprotection levels exceeding 95% in healthy infants, children, and young adults [23].

Universal vaccination programs have effectively reduced the global rate of chronic HBV infection to one-tenth of the pre-vaccination era. In Taiwan, for example, several epidemiologic surveys of serum HBV markers showed a significant decrease in HBsAg positivity, dropping from 10% to 0.6–0.7% after the introduction of vaccination [15,16,17] and even 0% in 3203 children aged 5–10 years in a recently study carried out in Colombia [24]. 

Similar patterns have been observed in Gambia and Korea. In Western countries, the incidence of HCC related to CHB has also declined since the 2000s, thanks to national vaccination programs and recommendations [25]. As a result, the incidence of CHB infection among children under five years old is now low, primarily attributed to the implementation of universal neonatal HBV vaccination programs.

One of the primary etiologic factors leading to HCC is HBV, highlighting the significant role of primary prevention from childhood to early adulthood. A 20-year follow-up study in Taiwan demonstrated that the HCC incidence was markedly lower among vaccinated children compared to those in unvaccinated birth cohorts (35.9%). Furthermore, among those who were vaccinated, the development of HCC was statistically associated with an incomplete HBV vaccination schedule [21].

However, while the aforementioned strategy for assessing HCC related to HBV is crucial, a population-level HBV vaccination program is expected to have a limited impact over the next two or three decades. Thus, a significant risk persists among those born before the vaccine became available. In these cases, secondary prevention through antiviral agents has emerged as a vital approach to reducing the short-term incidence of HCC. Currently, approved treatments include nucleos(t)ide analogs and interferons [26].

Some current practice guidelines fall short of addressing HCC prevention in cases without inflammation or liver fibrosis. This underscores the importance of increasing awareness among healthcare providers regarding the risk of HBV-associated hepatocarcinogenesis, which can occur independently of fibrosis [26].

In the case of nucleos(t)ide analogs, both Entecavir and Tenofovir Disoproxil Fumarate (TDF) have demonstrated similar efficacy in reducing HCC rates related to HBV, as shown in historical cohort studies. However, a recent meta-analysis involving 42,939 patients from Korea, Taiwan, and Hong Kong, alongside several smaller meeting reports, has sparked a debate on differing HCC rates under Entecavir (ETV) and Tenofovir Disoproxil Fumarate (TDF) treatment by favoring the latter, as TDF-treated patients had a significantly lower risk of developing HCC compared to ETV-treated patients [27]. Tenofovir Alafenamide (TAF), a more recent introduction, has also shown promising results. However, it requires further investigation due to the absence of long follow-up data [1].

Another preventive measure includes nucleos(t)ide treatment for highly viremic mothers during pregnancy. Tenofovir is preferred for its efficacy, safety in pregnant women, and low resistance rates. In a well-controlled prospective trial involving pregnant women in Taiwan, Tenofovir treatment reduced HBsAg positivity in infants, decreasing the prevalence from 10.71% to 1.54% [28].

In the case of HBV, liver cancer prevention can be categorized into three levels: primary, secondary, and tertiary [15]. Primary prevention is performed through universal HBV vaccine programs, aiming to prevent both the mother-to-child and horizontal transmission of HBV infection and representing the safest and most effective approach to preventing liver cancer [15,16,17]. Secondary prevention centers on patients with chronic hepatitis B by using antiviral agents to reduce viral load and then liver injury and fibrosis, as indicated by markers such as the normalization of transaminases [15,16,17]. Finally, tertiary prevention includes patients who have successfully undergone HCC treatment for whom antiviral agents are used to prevent HCC recurrence [15,16,17]. However, there remains a need to further reduce the risk of HCC by managing modifiable factors, including metabolic syndrome, aflatoxin exposure, heavy drinking, smoking, and other comorbidities that can contribute to liver inflammation. Equally crucial is the emphasis on minimizing high-risk behaviors related to blood and injection safety, not only because of their added risk to CHB, but also because they can independently lead to end-stage liver disease.

## 4. Hepatitis C Virus (HCV)

Another significant contributor to liver disease is hepatitis C. According to the Polaris Observatory Collaborators, over 56.8 million people worldwide are living with chronic hepatitis C, resulting in more than 400,000 deaths each year [29,30].

The prevalence of hepatitis C varies significantly by geographic location. The highest rates of infection are observed in low- and middle-income countries in Africa and Asia. Mongolia, for example, has a prevalence of over 4%, while in the Eastern Mediterranean region, it is close to 2%; in Europe, nearly 1.5%; and in the Western Pacific region and the Americas, the estimated prevalence is less than 1% [29].

The modes of HCV transmission vary based on regional factors and risk profiles. In high-income countries, the primary route is through injecting drug use, whereas in low-income countries, transmission often occurs through contaminated medical procedures and blood transfusions. Other potential avenues of transmission include unprotected sexual contact and mother-to-child transmission during childbirth [31].

As with HBV, it is imperative to implement effective preventive measures for HCV, not only due to the possibility of chronic infection, but also of the risk of developing liver tumors. In HCV patients, the incidence of HCC can increase by 10–20-fold [32], being responsible for approximately 30–50% of HCC cases worldwide [33]. Moreover, despite the changes in the epidemiology of HCC over the years, with metabolic-associated fatty liver disease gaining prominence, chronic hepatitis C remained the second leading cause of liver transplantation in the United States for men in 2019 and the third leading cause for women [34].

According to the natural history of HCV infection, up to 80% of individuals do not achieve spontaneous viral clearance, and in 20% of those patients, it progresses to cirrhosis [35]. Chronic hepatitis C often remains asymptomatic for several years, leading to a delay in diagnosis and treatment. Patients typically seek clinical care when symptoms related to complications of cirrhosis or HCC itself become evident, accounting for approximately 15% of cases [36].

Direct acting antiviral (DAA) therapy has revolutionized the treatment of HCV infection, offering a cure for most patients. Combinations of two (Sofosbuvir/Velpatasvir, Glecaprevir/Pibrentasvir) or three (Sofosbuvir/Velpatasvir/Voxilaprevir, used in treatment failure) DAAs have consistently achieved an overall SVR rate exceeding 95%. These treatments offer excellent tolerability and safety, which previous therapies did not, irrespective of factors such as genotype, fibrosis stage, intravenous drug abuse, or psychiatric comorbidities [32].

Attaining SVR through DAA therapy is strongly associated with a reduced risk of developing HCC, making SVR a pivotal factor in decreasing HCC incidence. Some meta-analyses of DAAs, especially pan-genotypic ones, have shown that achieving SVR can reduce the HCC risk by 50–80% [37]. It is essential to recognize that the risk of HCC does not return to baseline levels, particularly for patients with characteristics that are less favorable for achieving SVR [38].

As a result, international guidelines recommend post-SVR surveillance, including liver imaging and alpha-fetoprotein (AFP) tests every six months for cirrhotic patients. EASL guidelines extend this recommendation to individuals with advanced fibrosis (F3). The risk factors for post-SVR HCC development include age, male gender, lower baseline albumin, higher bilirubin levels, an FIB-4 score > 3.25, hepatitis B coinfection, and liver stiffness post-SVR ≥ 20 kPa [39].

Some studies have examined the impact of SVR achieved with DAAs on hepatic fibrosis in patients with chronic hepatitis C. These studies found that fibrosis improved by at least one stage in 56% of patients after a 15-month follow-up, and, notably, cirrhosis reversed in 29% of patients [40].

Another crucial consideration is the identification of surrogate markers predictive of fibrosis. For instance, splenomegaly was found to be a negative predictor of fibrotic improvement in cirrhotic patients who achieved SVR, in contrast to a platelet count greater than 152 × 10^9^/L, which served as a sensitive and specific marker for fibrosis regression [39]. It is essential to recognize the potential for fibrosis reversal, particularly in high-risk patients (those with advanced liver fibrosis—F3 or cirrhosis—at the time of DAA treatment). Doing so can lead to improved clinical outcomes, including a reduction in hepatic decompensation and complications, and a decreased risk of HCC development [40].

Preventive strategies for HCC in patients with HCV also include lifestyle modifications, such as reducing alcohol consumption and maintaining a healthy weight, which can help reduce the risk of HCC in these patients. Additionally, the management of comorbidities such as diabetes can also help reduce the risk of HCC [32].

Screening for HCC in patients with HCV is of utmost importance for early detection and treatment. Currently, ultrasound (US) serves as the established surveillance modality and is acknowledged as the most suitable imaging technique for HCC surveillance according to all international guidelines [41]. Cost-effectiveness studies have demonstrated that ultrasound-based surveillance every 6 months enhances quality-adjusted life expectancy at reasonable costs.

The early detection of HCC allows for curative treatments, such as resection, liver transplantation, or regional therapies, all of which significantly improve outcomes in patients with HCV [42].

In conclusion, preventing HCC in patients with HCV infection is crucial for reducing the global burden of liver cancer and its impact on communities. The effective, cost-efficient treatment of HCV, increased investment in screening and diagnosis, and lifestyle modifications all represent powerful strategies for preventing HCC in these patients. Further research is essential to enhance outcomes and alleviate the HCC burden in HCV-infected individuals.

## 5. Prevention of Fatty Liver Disease

The prevalence of fatty liver disease has also increased significantly in recent years. Most recent meta-analyses estimate the global prevalence for MASLD as being between 30% and 38% among the adult population [43]. As with any other chronic liver disease, MASLD, and particularly MASH, may also eventually lead to liver fibrosis in 35% of patients. Per year, approximately 2–5% of these patients will be diagnosed with liver cirrhosis [44]. Not surprisingly, MASLD is the fastest rising etiology of cirrhosis associated with acute-on-chronic liver disease (ACLF) among patients listed for liver transplantation in the US [45]. Also, of all patients with MASLD-derived cirrhosis every year, 2–3% will develop liver cancer [44].

However, fatty liver disease not only leads to cirrhosis, ACLF, liver cancer, and other liver-related complications. The disease is also closely linked to metabolic syndrome, and patients with fatty liver disease have a high rate of cardiovascular co-morbidities [46]. Most importantly, cohort studies clearly demonstrated that cardiovascular disease is the most common cause of death in patients with MASLD [47,48]. Also, in MASLD patients undergoing coronary angiography, the disease was significantly and independently correlated with the severity of coronary artery disease [49,50]. Furthermore, MASLD was repeatedly associated with an increased risk of stroke [51].

HCC development is a significant major threat to patients with MASLD. It is essential to acknowledge that MASLD-associated HCC may develop in patients with or without cirrhosis and that the rate of HCC in non-cirrhotic patients may be higher compared to patients with other chronic liver diseases, e.g., chronic viral hepatitis C. As these patients may not receive a standard surveillance ultrasound very six months, the diagnosis of these cancers may be significantly delayed, further aggravating the risk of a lethal course of the disease and considerably impacting the 5-year survival [52].

Generally, a cure for the disease could be fairly easy, as a body weight reduction of 7–10% improved liver fat content, MASH, and fibrosis [53]. Furthermore, intervention studies repetitively demonstrated the efficacy of weight loss in managing metabolic parameters and fatty liver disease [54]: blood pressure, insulin resistance, and muscle strength, among others, are significantly improved with weight loss, factors that are closely linked to metabolic syndrome [55].

However, weight loss is challenging for most patients, and long-term weight loss maintenance remains highly difficult. Some reports have reported most of the weight being regained after five years by a considerable number of patients [56,57].

At the same time, the medical treatment of MASLD also remains surprisingly challenging and, currently, no drugs are available or approved for the medical treatment of the disease. Just recently, a randomized phase III clinical trial of Resmetirom, targeting the thyroid hormone receptor (THR)-b, demonstrated positive effects on the reduction in fat content and fibrosis of the liver [58,59]. With these data, it is hoped that this drug will finally receive FDA approval. A second drug, obeticholic acid—a farnesoid X receptor (FXR) agonist—did not receive FDA approval despite positive phase III data [60].

With the lack of specific treatment options for MASLD, more unspecific treatment options primarily aimed at weight loss have become popular among overweight patients and patients with fatty liver disease. GLP-1 antagonists such as Semaglutide were demonstrated and approved for weight loss [61]. After FDA approval, the drug gained high popularity and was even temporarily sold out. Although GLP-1 may aid in weight loss efforts, it must be stressed that, according to the current data from a randomized phase II trial in patients with MASH and compensated cirrhosis, Semaglutide did not significantly improve fibrosis or achieve MASH resolution versus placebo [62].

In the absence of therapeutic drug options, bariatric surgery currently remains one of the main treatment options. Several surgical strategies were established, of which Roux-Y Gastric Bypass (RYGB) and Sleeve Gastrectomy are presently the most commonly applied procedures. No matter what procedure is used, bariatric surgery leads to effective weight loss in most overweight and fatty liver disease patients, but also reduces the cardiovascular risk and, ultimately, even patient mortality [63].

Overall, given the high prevalence of patients with MASLD, currently estimated to affect approximately 30% of the general population around the globe, its considerable impact on liver failure and carcinogenesis, improvements in the awareness and prevention of fatty liver disease have become a key issue in public health in most countries. With the current lack of available drugs, bariatric surgery should be considered in patients with advanced MASH and the failure of sufficient weight loss, as, in general, a weight loss of approximately 10% was demonstrated to be associated with a significant improvement in fatty liver disease.

## 6. Secondary Prevention of Esophageal Bleeding in Patients with Liver Cirrhosis

Even after the development of liver cirrhosis, preventative measurements may be of high clinical benefit for patients. Esophageal variceal bleeding because of portal hypertension may be prevented, or at least significantly reduced, through non-selective beta-blocker (NSBB) treatment.

The use of NSBBs in the primary prevention of esophageal bleeding is well established. By reducing cardiac output and splanchnic vasoconstriction resulting in a decrease in portal collateral blood flow [64], NSBBs were repetitively shown to be effective in both the primary and secondary prevention of esophageal bleeding [65,66,67]. In recent years, carvedilol was favored over propranolol [68], as an intrinsic anti-a1 adrenergic effect was demonstrated to cause intrahepatic vasodilatation and an additional decrease in portal pressure [64]. Thus, the most recent Baveno VII guidelines recommend carvedilol as the preferred NSBB in compensated cirrhosis, being more effective at reducing HVPG, providing better tolerability, and improving survival in compensated patients with clinically significant portal hypertension [69]. The use of NSBB in decompensated cirrhosis remains controversial. Based on initial data and trials discouraging NSBB use in decompensated cirrhosis, they are currently not recommended in many guidelines. However, several recent studies have questioned that dogma and provided evidence that a cautious use of NSBB may also be feasible and potentially effective in decompensated cirrhosis [70,71,72,73].

Alternatively, endoscopic band ligation (EBL) is currently thought to be equally effective in the primary prevention of first esophageal bleeding in patients with (high-risk) varices, although the data basis for their evaluation is somewhat heterogeneous. First, EBL had to be compared against NSBB treatment as they were already established by the time EBL became available. Several randomized controlled trials have shown a benefit of EBL in preventing first variceal hemorrhage. However, this effect was not visible in larger trials with more than 100 patients and longer follow-ups. Summarizing the available evidence, a Cochrane analysis found a beneficial effect of EBL on the primary prevention of upper GI bleeding in patients with esophageal varices. However, this effect did not impact mortality [74]. As it is currently assumed that EBL and NSBBs are equally effective, it is important to point out that NSBBs, in particular carvedilol, have a lower risk of serious complications compared to EBL [75,76].

Having established a potential role of EBL in the prevention of variceal bleeding, the use of both NSBBs and EBL is clinical routine in many hepatological centers around the globe. This leads to the obvious question of whether a combination of both treatment options would further increase the efficacy of bleeding prevention. Current data remain inconclusive, with some studies supporting a reduced probability of first bleeding [77], others stating a lower recurrence of varices if propranolol is added to EBL [78], and additional ones with data concluding that a combination does not add any benefit for the patient [79].

## 7. Prevention of Liver Cancer—HCC Surveillance

As previously stated, patients with liver cirrhosis have a significantly higher risk of developing HCC. Depending on the underlying disease, up to 6.5% of patients develop HCC each year [80]. Other risk constellations associated with an increased HCC rate are chronic hepatitis B and advanced fibrosis and cirrhosis. This significantly increased incidence justifies the regular surveillance of these patients using ultrasound or other imaging such as computed tomography (CT) or magnetic resonance imaging (MRI) [81]. Most recently, a large meta-analysis including almost 150,000 patients showed that patients who receive regular surveillance (ultrasound every 6 months) have a longer overall survival period [82]. This is due to the earlier detection of tumors in early tumor stages, among other factors [82]. Thus, more patients can be referred to curative therapy. An independent study also recently showed that shortening the surveillance interval to 3 months did not improve survival in these patients. In terms of the sensitivity and specificity of early HCC detection, conventional ultrasound and CT were comparable, while MRI performed better [83]. However, as MRI resources remain limited in many areas and ultrasound is ubiquitously available, ultrasound currently remains the imaging method of choice. In this respect, surveillance using ultrasound or other imaging methods has found its way into the recommendations of various gastroenterology or hepatology societies [81,84].

In contrast, a benefit of biomarkers in the early detection of liver cancer is still highly controversial. Heterogeneous data and the resulting meta-analyses have so far not shown an advantage for the use of AFP/AFP-L3 in the early detection of HCC [85,86]. Combinations of several markers including AFP, such as the GALAD (gender, age, AFP-L3, AFP, des-gamma-carboxy prothrombin) factor, are currently being discussed and undergoing extended clinical evaluation [85,87]. A combination of imaging with laboratory chemical markers such as AFP has also not been shown to have an advantage or additional benefit [88].

## 8. Preventive Substances—Metformin, Aspirin, Coffee, Statins

Numerous publications have suggested the preventive effects of diverse drugs and nutrients on the decompensation of liver cirrhosis and the development of HCC. Although none of these preventive options are clearly recommended in clinical practice guidelines, cumulating data, mostly from retrospective data analyses, at least warrant further evaluation of their potential use. Among the most prominent examples is metformin, which is encouraged to be evaluated in patients with liver cirrhosis for HCC prevention by German HCC guidelines [81]. Also, accumulating evidence on coffee consumption resulted in a recommendation to encourage patients with chronic liver diseases to drink coffee in order to decrease liver-related mortality and HCC development by the EASL clinical practice guideline [84]. Other substances such as statins, acetylsalicylic acid (ASA), or vitamin D supplementation were also under evaluation and were reported to show efficacy in larger retrospective analyses. We recently summarized the accumulating evidence elsewhere [89]. Finally, decreased anti-oxidant capabilities were repetitively discussed for chronic liver diseases, and the supplementation of vitamin E has been suggested by some authors as a possible “scavenger” of oxidative stress products. However, the role of vitamin E supplementation remains controversial [90], particularly since some reports discussed an association with an increased risk of prostate cancer [91,92]. However, the successful implementation of some of these strategies may potentially lead to additional improvement in the prevention of HCC development in patients with liver cirrhosis.

## 9. Further Improving Prevention in Hepatology—Awareness, Risk Stratification, and Big Data

The development of several effective prevention concepts is certainly among the biggest success stories in hepatology. As previously stated, with effective vaccination strategies, new therapeutic options, nutritional interventions, and even preventative surgical procedures, chronic progression to liver cirrhosis and its associated complications can be avoided for many liver diseases. Most importantly, this saves the patient considerable suffering and also saves society significant costs in the treatment of these liver diseases and secondary complications or even liver transplantation. Despite the availability of these effective preventive tools, it is evident and undisputed that there is still significant room for improvement.

Essential to further improvements in prevention in hepatology must be to increase awareness of liver diseases, preventive possibilities, and the usefulness of (regular) testing. To illustrate this issue, we launched a public website for patients to inquire about their risk for liver disease. Among more than 117,000 participants, 50.7% were uncertain about their liver enzyme status. In addition, approx. 30% were unsure about their hepatitis B vaccination status [93]. Obviously, even for such a potentially essential preventative intervention like hepatitis B vaccination, awareness could be significantly improved.

Simultaneously, it has recently been shown by the German Hepatitis C Registry and others that if we are, in fact, to regularly test more patients (i.e., regular follow-ups), we will indeed improve liver health. In Germany, health insurance companies just recently agreed that testing for hepatitis B and C would be part of regular check-up tests at the age of 35. The effectiveness of these measurements was evaluated by the German Hepatitis C Registry. Out of the 13,000 patients tested, 52 people had previously unknown anti-HCV antibodies and 8 were even HCV-RNA-positive. Thus, the number of patients that needed to be screened was 262; narrowing the HCV screening to risk factors such as (previous) drug abuse, blood transfusion before 1992, immigration to Germany, and elevated ALT further reduced the number needed to be screened to 111 [94].

Broad screening approaches and the detailed analysis of the resulting Big Data in healthcare could further improve prevention in hepatology. As an example, we recently demonstrated that influenza vaccination in patients with alcoholic liver cirrhosis may lead to a significant improvement in survival using the large public electronic health record collection of the international OHDSI (Observational Health Data Sciences and Informatics) consortium [95]. However, we found surprisingly few data available on vaccinations other than hepatitis A and B. In a similar approach, these methods were also used to further validate the beneficial effects of metformin and aspirin, but also the link to poorer prognosis in patients receiving catecholamines in patients with alcoholic liver disease [96]. Given the rapidly increasing and available health-related data for chronic liver disease, the joint efforts of clinicians and medical informaticists could unravel multiple further preventive aspects, particularly for co-medications and co-morbidities in patients with chronic liver disease.

With the increasing identification of patients with elevated liver values at risk for chronic liver disease, it will be important to refer patients with an elevated risk of chronic deterioration, a lack of prevention or treatment options in general practice, or an acute course of the disease to specialized centers. Given the enormous number of patients and considering that the prevalence of fatty liver disease alone is nearly 30% of the adult population worldwide, the stratification of patients is becoming increasingly important. In addition, close networking between general practitioners and specialists will be essential in order to enable the optimal use of the health system’s limited resources [97].

Since most of the health consequences of liver disease result from progressive chronic disease and from progressive fibrosis/cirrhosis, determining the degree of fibrosis using liver biopsy or elastography has emerged as an effective selection criterion. However, since these are not ubiquitously available and—particularly biopsy—involve considerable logistical effort, serological markers have increasingly emerged and been validated in recent years, particularly regarding liver-related events and mortality.

The Fibrosis-4 (FIB-4) Index for Liver Fibrosis is currently preferred in most clinical evaluations, particularly because of the easy availability of the included parameters (i.e., age, platelets, and transaminases) [98]. The further development of integrated care concepts must now involve the validation of these care concepts by incorporating serological or elastography markers into the stratified care of liver diseases [97]. If such validation is successful, it will certainly lead to a much more efficient use of the health system’s available resources, ultimately also enabling an even broader rollout of the already available, highly successful tool to prevent and halt the progression of liver disease.

Finally, novel developments in MASLD, hepatitis B, and hepatitis D treatment may, in the near future, offer even more options for the effective prevention of liver cirrhosis and its associated complications. Resmetirom has demonstrated positive effects on fat content and fibrosis in MASLD and may soon become the first FDA-approved drug to treat the disease and prevent its progression to cirrhosis [58,59]. Furthermore, recent progress in early clinical trials and novel approaches for a cure of hepatitis B could eventually translate into effective treatment strategies and a reduction in the global hepatitis B prevalence [99]. Subsequently, this would lead to a significant reduction in associated liver cirrhosis. Finally, the successful introduction of Bulevirtide for hepatitis D treatment in some countries may also help to prevent the development of liver cirrhosis in hepatitis B/D co-infected patients. A global introduction and approval may further aid in the efforts made towards limiting disease progression [100].

## 10. Conclusions

The development of preventive strategies and treatments in hepatology over the past decades truly is a success story. However, despite this medical progress, the prevalence of chronic liver diseases is increasing, and currently as much as 30% of the global adult population is assumed to suffer from elevated liver enzymes and chronic liver disease. Thus, continuous testing in primary care and awareness campaigns to motivate patients to be tested are crucial for further improvements in prevention in hepatology. Primary prevention through universal vaccination was proven highly effective for hepatitis B and is of high impact, particularly in high-prevalence areas. With the large number of patients suffering from fatty liver disease, stratification will be necessary for secondary prevention, as those with viral, metabolic, or autoimmune diseases or with a higher fibrosis grade need more specialized treatment in order to prevent liver cirrhosis and its complications. In contrast, patients with low fibrosis and a high likelihood of fatty liver disease may very well undergo an attempt of weight loss under the guidance of their primary care physician. Therefore, the FIB-4 was established as a simple marker for estimating fibrosis load. Finally, HCCs are detected earlier through consistent surveillance using ultrasound and patients are treated curatively more frequently.

In conclusion, vaccination and the early identification of patients for further surveillance and early treatment, as well as effective patient stratification, may further improve prevention in hepatology, as effective preventive options are already available for many diseases.

## Data Availability

Not applicable.
